# The Natural Enemies of the Mosquito

**Published:** 1902-10-04

**Authors:** 


### THE NATURAL ENEMIES OF THE MOSQUITO.

Dr. Louis W. Sambon,[1] of the London School of Tropical Medicine, writing about the prophylaxis of intermittent fevers, discusses an aspect of the question which has been far too little considered, namely, the possibility of limiting the multiplication of the malaria-bearing mosquitos by encouraging the growth of their natural enemies. That much good is being done by the methods which are at present so much in vogue no one doubts, but the vastness of the number of the mosquitoes, and their enormous powers of multiplication, are such as to make the task of eradication, by direct attack, a well-nigh hopeless one where large areas have to be dealt with. The chief preventive measure against malaria is undoubtedly the drainage and cultivation of the swamps and meres which are the breeding grounds of the malaria-bearing mosquitoes. Such measures have always proved efficacious whenever they have been thoroughly carried out. But owing to economic or other causes the obliteration of the swamp is not always possible. The kerosene treatment of the anopheles' breeding ground, again, is practically impossible over wide areas of uncultivated land, while the routine administration of quinine to the malaria-bearing native population, so as to avoid theinfection of new broods of mosquitoes, is in most regions out of the question. Although by combining these methods with the use of mosquito netting good results are often obtained, anything which leaves the mosquito in possession of the field must be inoperative as a permanent means of eradicating the disease. "I have no doubt, however," says Dr. Sambon, "that sooner or later we shall find some plant that will prevent the breeding of mosquitoes, or some innocuous gnat which will displace the dangerous anopheles, or some other animal which will prey upon it and prove as successful as the ladybird (*Vedalia cardinalis*) with which Dr. Riley saved the great orange industry of California that was being destroyed by a scale insect, the *Icerya purchasi*. Already we know that the common duckweed will prevent the breeding of anopheles in small quiet pools by completely covering the surface of water, and we know that wherever several species of mosquito larvæ are found together in the same body of water one of them is usually greatly preponderant, the others being apparently ousted by the most successful." Dr. Sambon then goes on to give a long list of the enemies of the mosquitoes, showing that even in the gnat tribe the larvæ of some kinds prey upon the larvæ of others. He also shows how the mosquitoes themselves are attacked by many parasites, of which, indeed, the malaria organism is one, and urges that should we fail to destroy the mosquitoes by means of their natural enemies there would still be hope of finding some hyper-parasite that might prevent the development of the parasites of intermittent fever within the body of the mosquitoes. Thus it might be possible, did we but know more about the intimate life and associates of the anopheles, to find some means of limiting its development more in accordance with Nature's methods than those at present in use. Lest however we should imagine that Nature's methods are always innocuous he instances the results of the introduction of the sparrow into the United States and the mongoose into Jamaica to inculcate caution, and to warn us that sometimes the benefits derived by such proceedings are far outweighed by their evil consequences.

[1] Journal of Tropical Medicine, Sept. 15

